# 

**Published:** 1922-09

**Authors:** 


### GOVERNMENT PUBLICATIONS.

[Copies can be purchased through any bookseller or direct from H.M. Stationery Office, at Imperial House, Kingsway, W.C.2; 28 Abingdon Street, S.W.1; 37 Peter Street, Manchester; 23 Forth Street, Edinburgh; and 1 St. Andrew's Crescent, Cardiff. The prices quoted include postage.]

Registrar-General, England and Wales. Annual Report (83rd), 1920. £1 1s.

Ministry of Health:—
- Third Annual Report, 1921-22. 6s. 3d.
- Annual Report of the Chief Medical Officer, 1921. 1s. 8d.
- Maternity and Child Welfare Memo. 65/M.C.W. Training of Health Visitors. 1½d.
- Model Bye-laws XVIII. Means of Escape from Fire in the case of certain Factories and Workshops. 6½d.
- Model Bye-laws VI. Slaughter-houses. 4d.
- Report of the Committee on Administration of Public Mental Hospitals. 3s. 2d.

Scottish Board of Health.—Report on the Administration of the Sale of Food and Drugs Acts and Abstract of Reports of Public Analysts for the year to Sept. 30, 1921. 3s. 1d.

### PARLIAMENTARY QUESTIONS AND ANSWERS.

July 19.—Sir Alfred Mond (to Mr. Robert Richardson): Under Section 49 of the Lunacy Act, 1890, the Board of Control has full discretion to authorise any medical practitioner to visit a patient for the purposes of the section. In the exercise of their discretion the Board endeavour to select medical practitioners who are in their opinion best qualified to act. The mere fact that the Board has not selected any particular medical practitioner whose name has been submitted to them is in no way defamatory, and there neither has been nor is any reason for consulting the Law Officers of the Crown in the matter.

Sir Alfred Mond (to Sir John Leigh): Tests are made from time to time to ensure that prescriptions are correctly dispensed, and severe penalties have been imposed in cases where chemists have supplied drugs of a kind different from that ordered by the doctor. If the hon. member will give particulars of any cases in which such statements [that insured patients can have superior medicines by paying for them] have been made, whether by practitioners or chemists, which have been brought to his notice, I will have inquiry made with a view to disciplinary action if the facts can be established.

July 26.—Sir A. Mond (to Sir W. de Frece): I am aware of the conference proposed to be held at Oldham [on smoke abatement], and have arranged that a representative of the Ministry shall attend. I shall be glad to encourage conferences on this subject where they are likely to serve a useful purpose.

Sir A. Mond (to Mr. Myers): I regret that I have no figures available as to the number of prosecutions instituted in England and Wales in respect of the adulteration of milk. In 1921 the number of samples of milk certified as adulterated or not up to standard was 5,290 out of 61,439 samples taken, compared with 5,533 out of 52,304 in 1913. I am unable to give separate figures for the different forms of adulteration.

Aug. 2.—Sir Alfred Mond (to Sir Robert Newman): So far as the Board of Control are aware, there are 58 county and borough mental hospitals which have no women members on the visiting committee. In these institutions there are approximately 31,000 female patients.

Aug. 3.—Sir Alfred Mond (to Mr. T. Thomson): The total sums disbursed as health insurance benefits and the total costs of administration during the last two years for England and Wales are as follows:—

| | 1920. | 1921. |
|---|---|---|
| Total expenditure on benefits ... | £18,782,000 | £21,562,000 |
| Total cost of administration ... | 4,500,000 | 4,827,000 |

Sir Alfred Mond (to Sir W. de Frece): My attention has been called to press reports of the statement [at Bournemouth, on Spray Disinfection]. The opinion of my advisers on the point is expressed in the memorandum on prevention of influenza issued by the Local Government Board in 1919, which states that the practice of spraying halls and places of public resort with a disinfectant fluid is of doubtful utility, and only tends to create a false sense of security.

Sir A. Mond (to Sir W. de Frece): The total number of scarlet fever cases reported in the first six months of 1922 was 54,966, and in the corresponding period of 1921, 56,023. No increase is shown in the more recent returns, the figure remaining fairly constant at about 2,000 weekly. Notification of measles is not now compulsory throughout England and Wales, and consequently no reliable figures can be given.

### PROGRESS OF LEGISLATION.

The following Bills have received the Royal Assent:—

Local Government and other Officers' Superannuation Bill.

Milk and Dairies (Amendment) Bill.

National Health Insurance Bill.

A second reading has been given to the Smoke Abatement Bill in the House of Lords, where it was introduced.

---

Despard House, in Currie Street, Nine Elms, has been presented by Mrs. Despard, who lived there for thirty years, to the Battersea Council for maternity and infant welfare work.

### RECENT APPOINTMENTS.

### Public Health.

Miss B. H. Poole, L.D.S.(Manch.), B.Sc.(Lond.), to be Dental Surgeon to the Rhondda Urban District Council.

Dr. W. A. Muir, M.D., Ch.B., D.P.H., at present Deputy M.O.H. for Wallasey, to be Medical Officer of Health, School Medical Officer and Medical Superintendent of the Isolation Hospital for the Borough of Gillingham, Kent.

### Institutional.

Viscount Lascelles has been appointed chairman of the Local Voluntary Hospitals Committee for the East and West Ridings of Yorkshire.

Dr. R. W. Locke, late Resident Medical Officer at the Maternity Hospital, New Bridge Street, Newcastle, to be Resident Medical Officer at Newcastle Dispensary.

Mrs. Wightwick, who lately resigned the post of honorary secretary to the Cobham and District Cottage Hospital after 10 years' work, has been succeeded by Mrs. Guy Morrish.

The new secretary and treasurer of Tewkesbury Hospital is Mr. Walter Derbyshire, who fills the post that Mr. Ralph Chandler had held for many years. Mr. Derbyshire has spent most of his life as a banker in the town, and has lately retired from the Tewkesbury branch of Lloyd's after 40 years' work. In addition to his professional duties, he has done much disinterested work as secretary, and finally Grand Master, of the Gloucester Conservative Benefit Society, as a Freemason, and as a lay-reader. Before the art, science, and technical classes were attached to the secondary schools, he was their honorary secretary, and similarly served the committee that organised the Gilchrist lectures. As a lay-reader, he has often held services at the Poor Law institution. We have often observed that local solicitors and bankers become attached to hospitals, and are generally the men who do the organising work for the scientific or art societies in their neighbourhood. Next year is Tewkesbury Hospital's diamond jubilee, so Mr. Derbyshire has watched it almost from its start.

### OFFICIAL REPORTS RECEIVED.

**(Year to Dec. 31, 1921, unless otherwise stated):—**

The Nightingale Fund. Year to December 25, 1921.

Association of Certificated Blind Masseurs. Year to June 30, 1922.

Borough of Middleton. Annual Report by Dr. S. Thos. Beggs, M.O.H. and S.M.O.

### Hospitals and Homes.

Middlesex Hospital, Mortimer Street, W.1.

Royal Victoria Infirmary, Newcastle-upon-Tyne.

Cancer Charity of the Middlesex Hospital, Nassau Street, W.1.

Gloucestershire Royal Infirmary and Eye Institution, Gloucester.

Carmarthen, Cardigan and Pembroke Joint Counties' Mental Hospital, Carmarthen.

Brighton County Borough Mental Hospital, Hayward's Heath.

---

The headquarters of the League of Red Cross Societies are now at 7 Rue Quentin Bauchart, Paris (VIII.), to which all correspondence and publications should be sent.

A National Milk Conference will be held on October 16, 17 and 18, in the Council Chamber of the Guildhall, London, by kind permission of the Corporation of the City of London, when papers will be read by experts on various aspects of the milk question and discussions will follow. Further particulars can be obtained from Miss H. M. Willans, Hon. Secretary, National Milk Conference, 3 Bedford Square, London, W.C.1.

All applicants who have been approved for registration, including those approved at the meeting of the General Nursing Council on September 22, 1922, will be qualified to vote for the election of the new Council in December, 1922. The names of nurses approved later than July 21, 1922, cannot appear in the first printed Register, but will be embodied in subsequent editions of the Register. All those approved for registration on September 22, will be notified of the fact.

